# Comorbidity in incident osteoarthritis cases and matched controls using electronic health record data

**DOI:** 10.1186/s13075-023-03086-8

**Published:** 2023-07-04

**Authors:** Anne Kamps, Jos Runhaar, Maria A. J. de Ridder, Marcel de Wilde, Johan van der Lei, Weiya Zhang, Daniel Prieto-Alhambra, Martin Englund, Evelien I. T. de Schepper, Sita M. A. Bierma-Zeinstra

**Affiliations:** 1 Department of General Practice, Erasmus MC, Dr. Molewaterplein 40, 3015 GD Rotterdam, Netherlands; 2 Department of Medical Informatics, Erasmus MC, Dr. Molewaterplein 40, 3015 GD Rotterdam, Netherlands; 3 School of Medicine, Faculty of Medicine & Health Sciences, Queen’s Medical Centre, University of Nottingham, Nottingham, NG7 2HA UK; 4 Nuffield Department of Orthopaedics, Rheumatology and Musculoskeletal Sciences (NDORMS), University of Oxford, Windmill Road, Headington, OX3 7HE Oxford UK; 5 Clinical Epidemiology Unit, Orthopaedics, Department of Clinical Sciences, Lund University, Wigerthuset, Remissgatan 4, 22185 Lund, Sweden; 6 Department of Orthopaedics and Sports Medicine, Erasmus MC, Dr. Molewaterplein 40, 3015 GD Rotterdam, Netherlands

**Keywords:** Osteoarthritis, Comorbidity, Primary Care, Epidemiology

## Abstract

**Background:**

Comorbidities are common in patients with osteoarthritis (OA). This study aimed to determine the association of a wide range of previously diagnosed comorbidities in adults with newly diagnosed OA compared with matched controls without OA.

**Methods:**

A case–control study was conducted. The data were derived from an electronic health record database that contains the medical records of patients from general practices throughout the Netherlands. Incident OA cases were defined as patients with one or more diagnostic codes recorded in their medical records that correspond to knee, hip, or other/peripheral OA. Additionally, the first OA code had to be recorded between January 1, 2006, and December 31, 2019. The date of cases’ first OA diagnosis was defined as the index date. Cases were matched (by age, sex, and general practice) to up to 4 controls without a recorded OA diagnosis. Odds ratios were derived for each 58 comorbidities separately by dividing the comorbidity prevalence of cases by that of their matched controls at the index date.

**Results:**

80,099 incident OA patients were identified of whom 79,937 (99.8%) were successfully matched with 318,206 controls. OA cases had higher odds for 42 of the 58 studied comorbidities compared with matched controls. Musculoskeletal diseases and obesity showed large associations with incident OA.

**Conclusions:**

Most of the comorbidities under study had higher odds in patients with incident OA at the index date. While previously known associations were confirmed in this study, some associations were not described earlier.

**Supplementary Information:**

The online version contains supplementary material available at 10.1186/s13075-023-03086-8.

## Background

Osteoarthritis (OA) is the most common form of arthritis and is ranked among the leading chronic diseases that cause pain and disability [1]. In addition, it is associated with all-cause mortality, as a recent meta-analysis reported that individuals with OA have an increased risk of premature mortality compared with those without (hazard ratios of 1.36–1.44) [2]. Over the last decades, the prevalence and incidence of OA have increased, and given the increasingly older and obese population, the prevalence is expected to continue to rise [3].

Since the prevalence of many other chronic conditions also rises with age, the frequent coexistence of OA with other diseases has in the past been attributed to aging only. However, a meta-analysis reported an age-adjusted difference in the prevalence of comorbidity among persons with (67%) versus without OA (56%) [4]. It also stated that individuals with OA were almost two times more likely to have multi-morbidity (≥ 2 comorbidities) than those of the same age without OA. The multifactorial disease mechanism and high prevalence of comorbidity indicate a complex relationship between OA, comorbidities, and their (shared) risk factors. To address the growing burden of OA, better knowledge of its associated comorbidities is required.

Several studies examined the association between OA and comorbidity, but many focused exclusively on one or a few comorbidities [5–9]. In addition, previous studies often grouped comorbidities, e.g., based on the affected organ, thereby losing essential information about different pathophysiology mechanisms per disease, as these may differ between diseases within the same organ. For example, thromboembolic and atherosclerotic diseases both affect the vessels but have distinguished pathways and risk factors.

In this study, the aim was to assess which long-term conditions, diagnosed by the general practitioner (GP), were associated with the diagnosis of OA. Comparing the odds of comorbidity in cases with incident OA to that in age-, sex-, and general practice-matched controls without OA can provide a better understanding of comorbidity patterns in OA and give insight into which disease mechanisms might contribute to the development of OA. A wide range of 58 comorbidities were investigated, several of which have been studied before, to validate the existing evidence on associations between OA and certain chronic conditions. At the same time, previously unexplored conditions were investigated to enable the generation of new hypotheses and encourage future (causal) research.

## Methods

An observational case–control study was conducted using data from the Integrated Primary Care Information (IPCI) database [10].

### Study source

IPCI is an electronic patient record database that was set up in 1996 and nowadays comprises medical records of more than 2.5 million patients from general practices throughout the Netherlands who are representative of the general Dutch population in terms of sex and age. In 2021, 1.4 of the 2.5 million were active patients, which corresponded to 8.1% of the Dutch population [11]. Patients entered the IPCI database by registering at one of the 350 participating practices. This could occur when the practice they were already registered with started participating in IPCI or when they moved to a new area and registered with a participating general practice there. IPCI data are collected until the date the most recent data extraction from the patient medical records took place, which is December 31, 2019 in this study. However, patients’ data collection may have ended at an earlier date when they died or deregistered or when their practice quit participating in IPCI.

IPCI contains longitudinal data on demographics, symptoms, diagnoses, test results, drug prescriptions (according to the Anatomical Therapeutical Chemical classification), referral to specialists, and hospital admissions [12]. The GP, who acts as a gatekeeper for referral to secondary care in the Netherlands, reports all relevant information obtained during consultation in the patient electronic record. In addition to text, the GP also registers a symptom, complaint, or diagnosis code according to the International Classification of Primary Care (ICPC) coding system [13]. The complete medical history is known for patients in IPCI since 1995, as most GPs started digitizing their records around that time. Codes of diseases that were diagnosed before digitization could still be added to the medical record in retrospect after asking patients for their medical history upon registration. At which date the corresponding diagnosis ICPC codes were registered differs: this can be the date on which patients registered in the practice or the dates in the past when the diagnoses were made.

A nested, matched case–control study was conducted. The IPCI database was used to form a study cohort with an observation period from January 1, 2006, to December 31, 2019. During this period, patients entered and exited the cohort depending on whether they met the criteria of potential cases and/or controls. To enter the cohort, at least 1 year of “active” IPCI database history and ≥ 18 years of age were required. Here, “active” meant that the diagnoses in this period were all prospectively recorded by the GP. Patients with an OA code recorded before January 1, 2006, were excluded because they were not at risk for the outcome incident OA. Patients exited the cohort at the final date of their available IPCI database history.

The scientific and ethical advisory board of the IPCI project positioned at the Erasmus MC Medical Center Rotterdam approved the study (registration number 11/2019).

### Selection of cases and controls

OA cases were defined as patients aged 18 or older with an incident OA diagnosis recorded in their medical records within the observational period. Diagnosis of OA was defined using the following OA ICPC codes: L89 (hip OA), L90 (knee OA), and L91 (other/peripheral OA). The first registration of an OA code within the observational period was defined as the case’s index date. Incidence density sampling (IDS) was performed to assign non-OA controls to each OA case, matched on age (± 2 years), sex, and general practice. Matching occurred at the index date, when a maximum of 4 of the available controls that met the case’s match criteria were selected at random. Selected controls received the same index date as the case to which they were matched. If there were less than 4 controls available, the next highest number of controls was selected, resulting in a 1:1–4 case–control ratio with optimal inclusion of participants. By selecting controls from the at-risk population, i.e., without prevalent OA at the index date but at risk to be diagnosed with OA afterwards, IDS provides less bias compared to regularly used survival sampling where controls remain disease-free throughout the study period. Therefore, this method is recommended for producing the least biased results of nested case–control analyses [14–16].

### Comorbidity

Various sources were consulted to select relevant, long-term comorbidities: diseases recommended by the European League Against Rheumatism for reporting comorbidity, the most prevalent and burdensome diseases according to the Global Burden of Disease study, mortality affecting diseases included in the Charlson comorbidity index, relevant diseases from a research tool for chronic conditions in primary care, and diseases that previously showed interesting associations in multimorbidity pattern studies were all considered [1, 9, 17–20]. This resulted in the selection of 58 comorbidities.

The diagnosis of each comorbidity was based on one or more corresponding ICPC codes. The referring ICPC codes had to be disease-specific. For example, diseases of the blood vessels were divided into “peripheral vascular disease,” “thromboembolic disease,” and “coronary artery disease,” all of which have different underlying disease mechanisms and risk factors. A second requirement was that the ICPC codes had kept a fixed definition throughout the course of the IPCI database. A full list of the comorbidities and their ICPC codes can be found in Supplementary Table 1.

### Statistical analysis

Of the total population in the cohort, only the successfully matched cases and controls were retained in the analysis set. Odds ratios (ORs) were calculated to compare the odds of each of the 58 prevalent comorbidities (exposure) between incident OA cases (outcome) and matched controls at the index date. In the main analysis, the entire IPCI database history of each person was examined for prevalent comorbidities, including the conditions that were registered before the observational period.

In addition, a sensitivity analysis was performed using only 1 year of database history prior to the index date, to identify the extent to which misclassification may have occurred and, if so, for which comorbidities this occurred. It was hypothesized that diagnoses that were recorded more often within 1 year prior to diagnosis of OA, compared to the entire available database history, could have been recorded for the same episode of complaints as OA was. This type of misclassification may have occurred in particular with comorbidities that are similar in presentation to OA, where the GP may have initially thought of another (musculoskeletal) disorder, but later made the working diagnosis of OA.

Since a matched case–control study design can introduce confounding by the matching factors, the ORs were controlled for the factors age and sex using unconditional logistic regression analysis [21]. A *P*-value of < 0.001 was used as the cut-off for statistical significance, to adjust for multiple testing. This value was derived from the standard *P*-value of < 0.05 after applying a Bonferroni correction for the 58 comorbidities studied and rounded to 3 decimal places for easier interpretation. To improve visual interpretability accordingly, each OR was displayed with 99.9% confidence interval (CI) error bars in the figures, so that it was clearly shown which associations were statistically significant different from 1. The statistical analyses were performed using SAS (software version 9.4) and R (software version 4.0.2).

## Results

After selecting the patients that met the criteria to become a case or control, the study population consisted of 1,890,712 individuals. During the observational period, 80,099 newly diagnosed OA cases were identified. Of these incident cases, most received a knee OA code (35,841 [44.7%]), followed by other/peripheral OA (22,231 [27.8%]) and hip OA (22,027 [27.5%]). 79,937 (99.8%) of them were successfully matched to 318,206 controls, which resulted in an analysis set of 398,143 persons. Baseline characteristics of the cases and controls are reported in Table 1.

**Table 1:** Baseline characteristics of analysis set

	**Cases** ***N* = 79,937**	**Controls** ***N* = 318,206**
**Age**^**a**^**, median (IQR)**	66.1 (57.4–74.8)	65.9 (57.3–74.6)
**Sex, percentage female**	64.0	64.0
**Observational period**^**b**^**, median (IQR)**	2.9 (1.3–4.9)	3.2 (1.5–5.3)
**BMI**^**c**^** in kg/m**^**2**^**, mean (SD)** *(available for 21% of analysis set)*	29.0 (5.6)	27.9 (5.9)
**Smoking status**^**c**^**, percentages** *(available for 37% of analysis set)*		
Current	19.6	29.0
Never	49.1	46.8
Past	31.3	24.2
**Alcohol**^**c**^** units per day, mean (SD)** *(available for 13% of analysis set)*	0.8 (1.6)	0.9 (1.7)

^a^Age in years was calculated at the index date

^b^Observational period in years counted from the start of the observational period until the index date

^c^The first recorded measure at, or after the start of the patients’ observational period was used. This corresponded best with the situation at baseline. Of BMI, smoking status, and alcohol use, there was only a limited availability of measurements in up to 37% of the analysis set

Patients with incident OA had higher odds of prevalence of 42 of the 58 comorbidities studied at the index date. The largest positive associations (ORs (99.9% CI)) were found for fibromyalgia 1.91 (1.68–2.16), obesity 1.79 (1.71–1.88), polymyalgia rheumatica 1.46 (1.21–1.76), spinal disc herniation 1.44 (1.40–1.49), and gout 1.40 (1.32–1.48).

For 13 conditions, no difference in odds for prevalent comorbidity between OA cases and controls was found. Lower odds in OA cases was found for only 3 comorbidities: multiple sclerosis 0.66 (0.50–0.88), dementia 0.80 (0.71–0.91), and schizophrenia 0.86 (0.77–0.97).

**Fig. 1:**
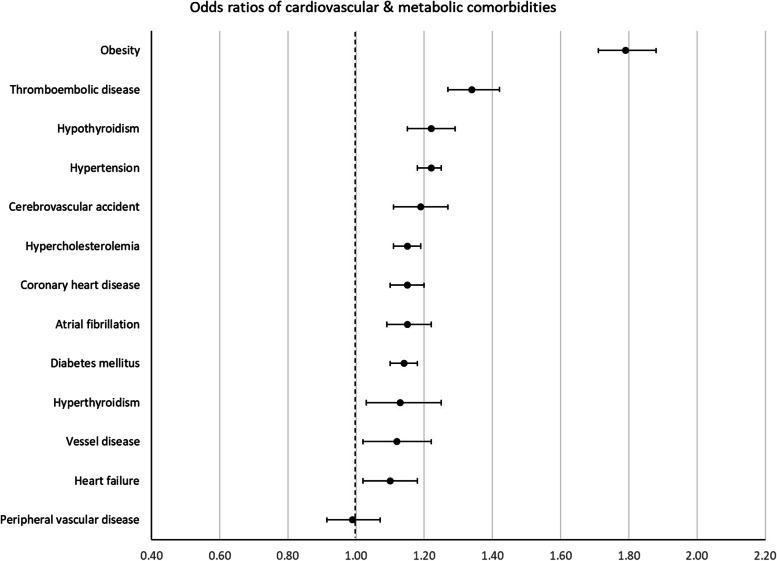
Odds ratios of cardiovascular and metabolic comorbidities

**Fig. 2:**
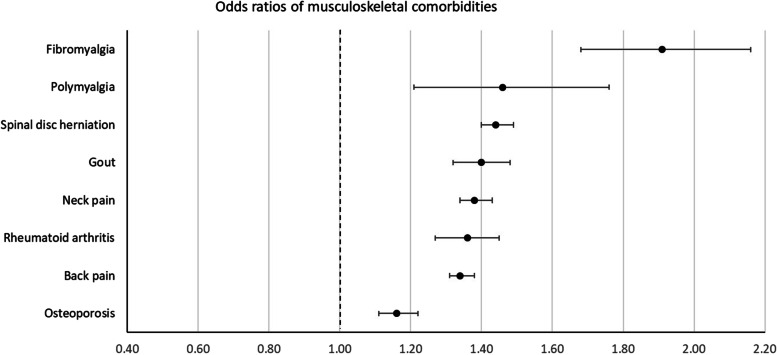
Odds ratios of musculoskeletal comorbidities

**Fig. 3:**
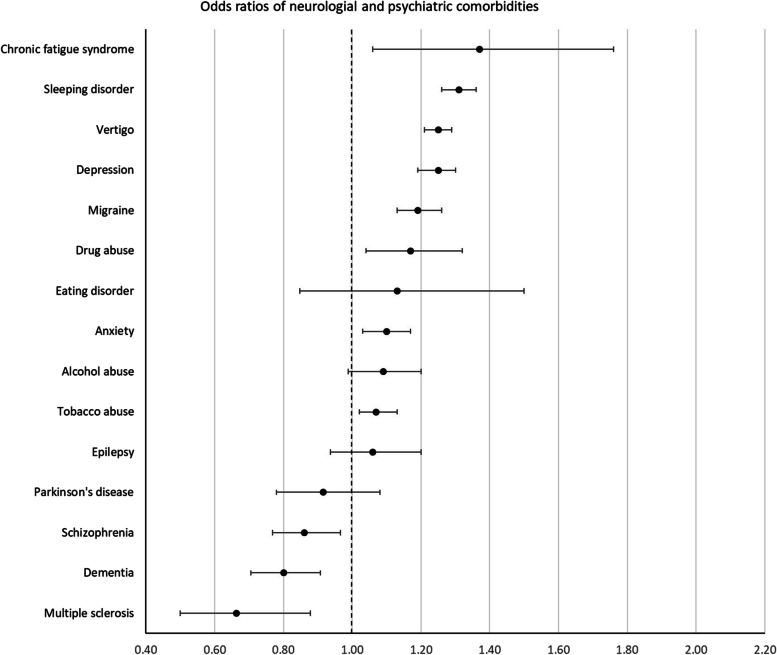
Odds ratios of neurological and psychiatric comorbidities

**Fig. 4:**
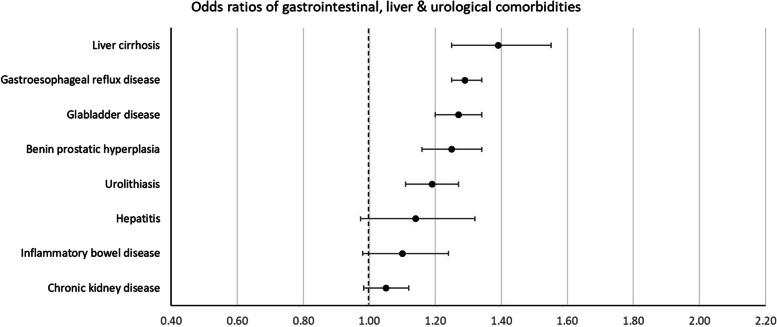
Odds ratios of gastrointestinal, liver, and urological comorbidities

**Fig. 5:**
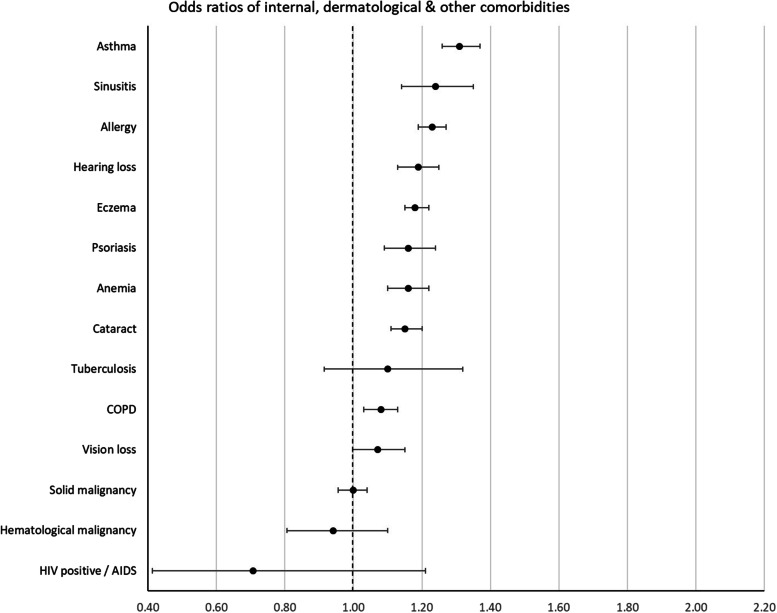
Odds ratios of internal, dermatological, and other comorbidities. COPD, chronic obstructive pulmonary disease

The ORs with 99.9% CI error bars of all 58 comorbidities are visualized in Figs. 1, 2, 3, 4, and 5, grouped per area of interest.

The sensitivity analysis showed 5 comorbidities that were diagnosed more often in cases than controls within 1 year before the index date: fibromyalgia, obesity, gout, rheumatoid arthritis, and spinal disc herniation (ORs ranging from 1.62 to 1.13 respectively). Twenty-one comorbidities showed no (statistically significant) difference. Thirty-two comorbidities showed ORs lower than 1, indicating these comorbidities were less diagnosed in OA cases than matched controls within one year before the index date. The smallest ORs were found for hematological malignancy 0.54 (0.38–0.77), solid malignancy 0.67 (0.61–0.74), epilepsy 0.68 (0.48–0.98), tobacco abuse 0.69 (0.61–0.78), and schizophrenia 0.70 (0.55–0.90).

The detailed results of both main and sensitivity analysis including the ORs with corresponding CIs and *P*-values are added in Supplementary Tables 2 and 3.

## Discussion

### Main findings

This study showed that the odds of having prevalent comorbidity differed between adults with newly diagnosed OA and their matched controls. Incident OA was positively associated with the majority (~ 72%) of the comorbidities, meaning that the probability of having these comorbidities was greater in individuals with incident OA than in individuals without OA at the index date, regardless of age, sex, and general practice.

For conditions that are well-known risk factors for OA, such as obesity, a higher OR was expected [22–26]. Based on previous studies, the association of OA with other musculoskeletal and joint diseases was also anticipated [27, 28]. In addition, other known associations were confirmed in this study, such as depression, gastroesophageal reflux disease, vertigo, and asthma [5, 7, 9, 28, 29]. Asthma, for example, was called a “novel association” by Koo et al. in a 2021 study. For depression, the association with OA has been repeatedly described. A common hypothesis is that depression and painful symptoms follow the same (biochemical) pathways of the central nervous system [30, 31].

Some positively associated comorbidities have not been described before, among which liver cirrhosis, chronic fatigue syndrome, thromboembolic disease, allergy, and migraine (ORs of 1.39, 1.37, 1.34, 1.23 and 1.19 respectively). These conditions provide interesting new leads for further investigation.

A possible explanation for ORs significantly lower than 1 that are seen in short-term fatal diseases can be found in the fact that OA may be subordinated (by both patient and physician) to such severe diseases and as a result be registered less frequently. Furthermore, certain psychiatric and neurological disorders such as dementia and schizophrenia can cause cognitive and/or communicative impairments (e.g., altered memory, pain perception, or expression) that might complicate the diagnosis of other subsequent diseases and therefore result in an OR below 1.

### Sensitivity analysis findings

The sensitivity analysis showed 5 conditions that were diagnosed more often in cases than controls within the year before the index date. Most of these comorbidities have a similar clinical presentation to OA or are strongly associated with OA. The fact that GPs can register multiple ICPC codes during an episode of complaints might partly explain this. A GP can register the code that represents the most likely diagnosis at the first consultation (e.g., fibromyalgia), but add another code (e.g., knee OA) on a follow-up visit when a different diagnosis becomes more likely, often after additional testing has been performed. Previously registered codes will remain in the medical record and will display as prevalent comorbidities in the year before OA diagnosis.

Most comorbidities showed no difference or were less diagnosed in OA cases compared with controls in the year before the index date. A hypothesis for ORs below 1 is that patients (and GPs) prioritize OA complaints shortly before OA diagnosis, resulting in less attention for other symptoms and therefore fewer diagnoses of certain comorbidities than the matched controls.

### Difference prior literature

Previous studies that examined comorbidities in OA found cardiometabolic diseases such as hypertension, hypercholesterolemia, and diabetes to be positively associated with OA. Metabolic syndrome, for example, a cluster with the aforementioned conditions and (central) obesity as main components, was about 2 times more prevalent in patients with OA [4]. Other meta-analyses reported ORs of 1.41 for diabetes and 1.37 for dyslipidemia [32, 33].

In the current study, hypertension, hypercholesterolemia, and diabetes were also positively associated with OA, but the effect sizes were smaller than in the abovementioned studies (ORs of 1.22, 1.15 and 1.14, respectively). The majority of previous studies estimated the association of comorbidities with OA at one time point across the entire population. Hence, the estimate cannot differentiate between the sequence of events, i.e., whether the diagnosis of comorbidity or of OA came first [34]. In this study, odds of comorbidity were estimated at the cases index date using IDS. Thus, the lower ORs might reflect the difference in study design.

### Strength

In case–control studies, the relative risk (RR) as obtained from cohort studies can be approximated by the OR, provided that the methodological requirements are met. In practice, the OR often exaggerates the RR, which can be caused by the selection of a biased, “over healthy” reference group [15, 28, 35]. This bias is mostly introduced by selecting controls that remain completely disease-free for the entire study period (“survivor sampling”). What distinguishes the current study from most previous case–control studies is that controls were selected from the entire at-risk population at the time of the case’s incident OA diagnosis (i.e., the index date) via IDS. Selection bias was prevented and therefore the ORs in this study estimated the true RRs without the requirement of the rare disease assumption [14, 15]. This is one of the study strengths.

### Limitations electronic health records

The use of electronic health records for epidemiological studies also comes with certain weaknesses. Little is known about the accuracy of diagnostic ICPC codes for their use in observational studies. For comparable coding systems such as Read codes and ICD codes, studies showed high specificity, low to moderate sensitivity, and moderate to good positive predictive values for diagnostic codes [36–39].

Besides the accuracy of ICPC codes, the coding behavior of GPs might influence the data as well. It is likely that the prevalence of comorbidity is underestimated using codified data alone. This is because not all GPs will register a diagnosis code but instead may write the diagnosis in free text or register a symptom ICPC code instead (“knee pain” instead of “knee OA”). It is assumable that certain diseases, especially those with many corresponding symptom codes or ambiguous diagnostic criteria, are disproportionally under recorded compared to very specific diseases with strict diagnostic criteria. To prevent potential differences in GP coding behavior and/or misbalance in under recording from having an effect on the results, cases and controls were matched by general practice in this study.

Surveillance bias may also have affected the data. Patients who frequently visit their GP—sometimes due to chronic conditions that require periodic check-ups—are likely to have more diagnoses registered. This is, among others, likely due to an increased risk of incidental findings and more frequent examinations that result from these visits. However, there is no convenient way to adjust for the frequency of GP visits in IPCI without inadvertently creating another bias for certain groups. Therefore, it must be considered that not all patients had the same chance of being registered with a certain disease code.

If there is an association between the matching factor and the exposure (prevalent comorbidity in this study), then matching might introduce confounding that requires controlling for the factor [21]. The matching factor “general practice” could not be adjusted for in the logistic regression due to the large number of practices. However, it was assumed that there was only little association between the exposure and this matching factor and therefore preferred not to correct for at all, instead of correcting through suboptimal alternatives (e.g., reducing the number of levels by combining practices).

### Confounders

Although potential confounders such as obesity, physical inactivity, and social determinants might have played a (mediating) role in the reported associations, adjusting for determinants other than age and sex was not performed in this study. No adjustments could be made for the factors BMI, smoking, and alcohol consumption due to the proportion of missing data in these variables within the IPCI database. Moreover, an inspection of the missing data revealed that these were not missing at random; thus, imputation would introduce severe bias. For example, GPs registered weight more often in people who appeared to be significantly overweight or in people who needed certain medications for which their weight was required for prescription. Furthermore, physical inactivity and social determinants of health, for example social economic status and educational level, were not included as variables in the IPCI database at all, but are widely accepted as important factors regarding morbidity and mortality [40].

### Broad definition

Finally, the definition of OA cases was general and based on the combination of the 3 available OA localization subtypes, as the main objective in this study was to look at OA as a whole and explore the common associated conditions. Using a broad definition, distinctive associations of comorbidities with knee versus hip OA could not be examined. Other/peripheral OA was a highly heterogeneous subtype that included multiple OA sites, including the hand and foot, and therefore could not be used to demonstrate site-specific associations with comorbidity.

## Conclusions

To conclude, this study showed that odds were higher for 42 out of 58 studied physician-diagnosed comorbidities in patients with newly diagnosed OA compared with age-, sex-, and general practice-matched controls. The study confirms known associations but also provides many new insights into comorbidity patterns. The higher odds for prevalent liver cirrhosis and asthma in patients with OA, for example, provide a starting point for further research into the underlying mechanisms and potential causal relationships of these associations.

Knowing which comorbidities are more common in OA patients is the first step in contributing to reducing the large burden of OA. The wide range of individually examined comorbidities and the strong sampling design make this study a unique and valuable addition to the growing body of evidence about comorbidities in OA.

## Supplementary Information

**Additional file 1:** **Supplementary Table 1.** Disease definitions: ICPC codes that were used to define osteoarthritis and each comorbidity.

**Additional file 2:** **Supplementary Table 2.** Results of the main analysis: prevalence (per 1000 persons), age & sex adjusted odds ratios with 99.9% confidence intervals and P-values of all comorbidities, assessed over the entire available medical history in the IPCI database.

**Additional file 3:** **Supplementary Table 3.** Results of the sensitivity analysis: age & sex adjusted odds ratios with 99.9% confidence intervals and P-values of all comorbidities, assessed within the period of 1 year prior to the index date.

## Data Availability

The data that support the findings of this study are available via the IPCI database but restrictions apply to the availability because it concerns individual patients’ electronic health record data, which were used under license for the current study, and so are not publicly available. Data might however become available upon reasonable request to the IPCI database board. The codes developed for the analysis will be available upon reasonable request.

## Abbreviations

OA: Osteoarthritis
OR: Odds ratio
IPCI: Integrated Primary Care Information
GP: General practitioner
ICPC: International Classification of Primary Care
IDS: Incidence density sampling
CI: Confidence intervals
RR: Relative risk
