# Supplementary material for: Comorbidity in incident osteoarthritis cases and matched controls using electronic health record data
Source: Arthritis Res Ther. 2023 Jul 4;25:114. doi: 10.1186/s13075-023-03086-8 (PMC10318652; doi:10.1186/s13075-023-03086-8)
Supplement: Supplementary file 1 — Additional file 1: Supplementary Table 1. Disease definitions: ICPC codes that were used to define osteoarthritis and each comorbidity. [file 13075_2023_3086_MOESM1_ESM.docx]

Supplementary table 1. Disease definitions: ICPC codes that were used to define osteoarthritis and each comorbidity

| Conditions | ICPC codes |
| --- | --- |
| 1. Anaemia | B78 Inheritable haemolytic anaemia  B78.01 Thalassemia  B78.02 Sickle cell anaemia  B78.03 Anaemia through G6PD-deficiency  B80 Iron deficiency anaemia  B81 Pernicious/folium acid deficiency-anaemia  B81.01 Folium acid deficiency-anaemia  B81.02 Vitamin B12-deficiency-anaemia  B82 Other/non-specified anaemia |
| 1. Anxiety | P74 Anxiety disorder / state of anxiety  P74.01 Panic attacks / disorder  P74.02 Generalized anxiety disorder |
| 1. Asthma | R96 Asthma  R96.01 Hyper reactivity airway  R96.02 Allergic asthma |
| 1. Benign prostatic hypertrophy | Y85 Benign prostate hypertrophy |
| 1. Atrial fibrillation | K78 Atrial fibrillation |
| 1. Cataract | F92 Cataract  F92.01 Senile cataract |
| 1. Back pain | L02 Back symptoms/complaints  L03 Low back-pain without radiation [ex. L86] |
| 1. Chronic kidney disease | U99.01 Renal impairment |
| 1. Neck pain | L01 Neck symptoms/complaints [ex. N01] |
| 1. Sinusitis | R75.02 Chronic sinusitis |
| 1. COPD | R95 Emphysema/COPD  R91 Chronic bronchitis/bronchiectasis  R91.01 Chronic bronchitis  R91.02 Bronchiectasis |
| 1. Coronary heart disease | K74 Angina pectoris  K74.01 Instable angina pectoris  K74.02 Stable angina pectoris  K75 Acute myocardial infarction  K76 Other/chronic ischemic heart disease  K76.01 Coronary sclerosis  K76.02 Past myocardial infarction (> 4 wks. ago) |
| 1. Dementia | P70 Senile dementia/Alzheimer  P70.01 Alzheimer  P70.02 Multi-infarct dementia |
| 1. Depression | P76 Depression  P76.01 Post-partum depression |
| 1. Diabetes mellitus | T90 Diabetes mellitus  T90.01 Diabetes mellitus type 1  T90.02 Diabetes mellitus type 2  F83.01 Diabetic retinopathy |
| 1. Hypercholesterolemia | T93 Lipid disorders |
| 1. Eating disorder | T06 Anorexia nervosa/bulimia  T06.01 Anorexia nervosa  T06.02 Bulimia |
| 1. Eczema | S86 Seborroic eczema/dandruff  S86.01 Dandruff  S86.02 Cradle cap  S87 Constitutional eczema  S88 Contact eczema/other eczema  S88.01 Contact eczema  S88.02 Dyshidrotic eczema  S88.03 Dermatitis after application local medicine  S88.04 Sun allergy/photo dermatosis |
| 1. Epilepsy | N88 Epilepsy (all types) |
| 1. Chronic fatigue syndrome | A04.01 Chronic fatigue syndrome |
| 1. Fibromyalgia | L18.01 Fibromyalgia |
| 1. Gall bladder disease | D98 Cholecystitis/cholelithiasis  D98.01 Cholecystitis  D98.02 Cholangitis  D98.03 Cholelithiasis without inflammation |
| 1. Gastroesophageal reflux disease | D84 Disease oesophagus  D84.01 Diverticula oesophagus  D84.02 Oesophageal reflux without esophagitis  D84.03 Oesophageal reflux with esophagitis  D84.05 Oesophagus stenosis  D85 Duodenal ulcer  D86 Other peptic ulcer  D86.01 Ventricular ulcer  D87 Stomach dysfunctional disorder  D87.01 Gastritis/duodenitis  D87.02 Dyspepsia/indigestion |
| 1. Gout | T92 Gout |
| 1. Hearing loss | H83 Otosclerosis  H84 Presbyacusis  H85 Acoustic damage/noise deafness  H86 Deafness/ hearing impairment |
| 1. Heart failure | K77 Congestive heart failure  K77.01 Acute congestive heart failure/asthma cardiae  K77.02 Chronic congestive heart failure |
| 1. Hepatitis | D72 Viral hepatitis  D72.01 Acute hepatitis A  D72.02 Acute hepatitis B  D72.03 Acute hepatitis C  D72.04 Carrier hepatitis B/chronic hepatitis B  D72.05 Carrier hepatitis C/chronic hepatitis C |
| 1. HIV positive / AIDS | B90 HIV-infection (AIDS/ARC)  B90.01 Seropositive without symptoms  B90.02 AIDS/ARC |
| 1. Hypertension | K86 Essential hypertension without organ damage  K87 Hypertension with organ damage/secondary hypertension  F83.02 Hypertensive retinopathy |
| 1. Hyperthyroidism | T85 Hyperthyroidism/thyrotoxicosis |
| 1. Hypothyroidism | T86 Hypothyroidism/myxoedema |
| 1. Inflammatory Bowel Disease | D94 Colitis ulcerative/chronic enteritis (regionalis)  D94.01 Colitis ulcerative  D94.02 Crohn’s disease |
| 1. Haematological malignancy | B72 Hodgkin’s disease  B72.01 Hodgkin’s disease  B72.02 Non-Hodgkin lymphoma  B73 Leukaemia  B74 Other malignancy blood/lymph  B74.01 Multiple myeloma |
| 1. Liver cirrhosis | D97 Cirrhosis/other liver disease  D97.04 Cirrhosis  D97.05 Liver steatoses |
| 1. Migraine | N89 Migraine |
| 1. Multiple sclerosis | N86 Multiple sclerosis |
| 1. Osteoarthritis | L89 OA of hip  L90 OA of knee  L91 other/non specified OA |
| 1. Osteoporosis | L95 Osteoporosis  L95.01 Osteopenia  L95.02 Osteoporosis |
| 1. Other blood vessel disease | K92 Other diseases peripheral arteries  K92.02 Raynaud’s syndrome  K92.03 Buerger’s disease |
| 1. Parkinson’s disease | N87 Parkinsonism, Parkinson’s disease  N87.01 Parkinson’s disease |
| 1. Peripheral vascular disease | K91 Atherosclerosis [ex. K76,K90]  K92.01 Intermittent claudication |
| 1. Polymyalgia rheumatica | L99.12 Polymyalgia rheumatica |
| 1. Psoriasis | S91 Psoriasis (with or without arthropathy) |
| 1. Rheumatoid arthritis | L88 Rheumatoid arthritis/associated conditions  L88.01 Rheumatoid arthritis  L88.02 Morbus Bechterew (ankylopoetic spondylitis)  K71 Acute rheuma/rheumatic heart disease  K71.01 Acute rheuma without heart disease  K71.02 Acute rheuma with heart disease |
| 1. Schizophrenia | P71 Other organic psychosis  P71.04 Delirium [ex.P15.02]  P72 Schizophrenia  P73 Affective psychosis  P73.02 Bipolar disorder  P98 Other/not specified psychoses |
| 1. Allergy | A12 Allergy/allergic reaction  A12.01 Anaphylactic shock  A12.02 Angioneurotic/Quinckes oedema  A85.01 Allergy for medication  R97 Hay fever/allergic rhinitis |
| 1. Sleeping disorder | P06 Insomnia/other sleeping disorder  P06.01 Sleep apnoea syndrome |
| 1. Solid malignancy | A79 Malignancy of unknown primary localization  D74 Malignancy stomach  D75 Malignancy colon/rectum  D76 Malignancy pancreas  D77 Other/non-specified malignancy digestive organs  D77.01 Malignancy oesophagus  D77.02 Malignancy salivary glands  D77.03 Malignancy lip/mouth/tongue  D77.04 Malignancy liver/gall bladder/bile duct  H75.01 Malignancy ear  K72.01 Malignancy heart/vessels  N74 Malignancy nerve system  R84 Malignancy bronchus/lung  R85 Other malignancy airways  S77.03 Malign melanoma  T71 Malignancy thyroid  U75 Malignancy kidney  U76 Malignancy bladder  U77 Other malignancy urinary tract  W72 Malignancy associated with pregnancy  X75 Malignancy cervix uteri  X76 Malignancy breast woman  X76.01 Adenocarcinoma mamma woman  X77 Other malignancy reproductive organ woman  X77.01 Endometrium carcinoma  X77.02 Malignancy ovarian  Y77 Malignancy prostate  Y78 Other malignancy reproductive organs/breast man  Y78.01 Malignancy penis  Y78.02 Malignancy testis  Y78.03 Malignancy breast |
| 1. Cerebrovascular accident | K89 TIA  K90 Cerebrovascular accident (CVA)  K90.01 Subarachnoid bleed  K90.02 Intracerebral bleed  K90.03 Cerebral infarction |
| 1. Drug abuse | P18 Medication abuse  P19 Drug abuse  P19.01 Abuse/addiction soft drugs  P19.02 Abuse/addiction hard drugs |
| 1. Thromboembolic disease | K93 Pulmonary embolism/lung infarction  K94 Thrombophlebitis  K94.01 Deep venous thromboses leg  K94.02 Superficial thrombophlebitis  W99.03 Thrombosis childbed |
| 1. Tuberculosis | A70 Generalized tuberculosis [ex. R70]  R70 Tuberculosis airways [ex. A70] |
| 1. Vertigo | H82 Vertigo syndrome/labyrinthitis [ex. N17]  H82.01 Meniere’s disease  H82.02 Labyrinthitis/vestibulitis  H82.03 Benign paroxysmal position vertigo  N17 Vertigo/dizziness [ex. H82]  N17.01 Vertigo  N17.02 Light headed |
| 1. Vision loss | F93 Glaucoma  F93.01 increased eye pressure without known glaucoma  F93.02 Acute glaucoma  F93.03 Glaucoma simplex  F93.04 Secondary glaucoma  F94 Blind (every grade/type) |
| 1. Obesity | T83 Obesity |
| 1. Spinal disc herniation | L83.01 Cervical spinal disc herniation  L86 Low back pain with radiation  L86.01 spinal disc herniation (thoracic/lumbar) |
| 1. Urolithiasis | U95 Urolithiasis |
| 1. Tobacco abuse | P17 Tobacco abuse |
| 1. Alcohol abuse | P16 Alcohol abuse / intoxication |
