# Supplementary material for: Comorbidity in incident osteoarthritis cases and matched controls using electronic health record data
Source: Arthritis Res Ther. 2023 Jul 4;25:114. doi: 10.1186/s13075-023-03086-8 (PMC10318652; doi:10.1186/s13075-023-03086-8)
Supplement: Supplementary file 2 — Additional file 2: Supplementary Table 2. Results of the main analysis: prevalence (per 1000 persons), age & sex adjusted odds ratios with 99.9% confidence intervals and P-values of all comorbidities, assessed over the entire available medical history in the IPCI database. [file 13075_2023_3086_MOESM2_ESM.docx]

Supplementary table 2. Results of the main analysis: prevalence (per 1000 persons), age & sex adjusted odds ratios with 99.9% confidence intervals and P-values of all comorbidities, assessed over the entire available medical history in the IPCI database

| Comorbidity | Prevalence cases | Prevalence controls | OR | Lower 99.9% CI | Upper 99.9% CI | P-value |
| --- | --- | --- | --- | --- | --- | --- |
| Fibromyalgia | 13.08 | 6.99 | 1.91 | 1.68 | 2.16 | <0.001 |
| Obesity | 96.29 | 56.75 | 1.79 | 1.71 | 1.88 | <0.001 |
| Polymyalgia rheumatica | 5.32 | 3.62 | 1.46 | 1.21 | 1.76 | <0.001 |
| Spinal disc herniation | 221.28 | 164.64 | 1.44 | 1.40 | 1.49 | <0.001 |
| Gout | 60.04 | 43.76 | 1.40 | 1.32 | 1.48 | <0.001 |
| Liver cirrhosis | 16.31 | 11.81 | 1.39 | 1.25 | 1.55 | <0.001 |
| Neck pain | 199.59 | 153.47 | 1.38 | 1.34 | 1.43 | <0.001 |
| Chronic fatigue syndrome | 2.91 | 2.14 | 1.37 | 1.06 | 1.76 | <0.001 |
| Rheumatoid arthritis | 42.50 | 31.55 | 1.36 | 1.27 | 1.45 | <0.001 |
| Back pain | 425.41 | 355.91 | 1.34 | 1.31 | 1.38 | <0.001 |
| Thromboembolic disease | 56.80 | 42.80 | 1.34 | 1.27 | 1.42 | <0.001 |
| Sleeping disorder | 112.02 | 88.11 | 1.31 | 1.26 | 1.36 | <0.001 |
| Asthma | 148.35 | 117.38 | 1.31 | 1.26 | 1.37 | <0.001 |
| Gastroesophageal reflux disease | 175.51 | 141.18 | 1.29 | 1.25 | 1.34 | <0.001 |
| Gallbladder disease | 68.50 | 54.85 | 1.27 | 1.20 | 1.34 | <0.001 |
| Vertigo | 42.12 | 34.35 | 1.25 | 1.21 | 1.29 | <0.001 |
| Depression | 106.57 | 87.73 | 1.25 | 1.19 | 1.30 | <0.001 |
| Benin prostatic hyperplasia | 221.14 | 185.70 | 1.25 | 1.16 | 1.34 | <0.001 |
| Sinusitis | 26.26 | 21.33 | 1.24 | 1.14 | 1.35 | <0.001 |
| Allergy | 201.82 | 171.72 | 1.23 | 1.19 | 1.27 | <0.001 |
| Hypertension | 404.73 | 360.29 | 1.22 | 1.18 | 1.25 | <0.001 |
| Hypothyroidism | 56.96 | 47.29 | 1.22 | 1.15 | 1.29 | <0.001 |
| Hearing loss | 68.20 | 64.73 | 1.19 | 1.13 | 1.25 | <0.001 |
| Cerebrovascular accident | 85.03 | 72.38 | 1.19 | 1.11 | 1.27 | 0.003 |
| Migraine | 61.21 | 52.43 | 1.19 | 1.13 | 1.26 | <0.001 |
| Urolithiasis | 42.20 | 35.77 | 1.19 | 1.11 | 1.27 | <0.001 |
| Eczema | 336.06 | 299.55 | 1.18 | 1.15 | 1.22 | <0.001 |
| Drug abuse | 11.86 | 10.15 | 1.17 | 1.04 | 1.32 | <0.001 |
| Anemia | 72.41 | 63.01 | 1.16 | 1.10 | 1.22 | <0.001 |
| Osteoporosis | 82.01 | 71.24 | 1.16 | 1.11 | 1.22 | <0.001 |
| Psoriasis | 42.39 | 36.65 | 1.16 | 1.09 | 1.24 | <0.001 |
| Hypercholesterolemia | 56.96 | 49.52 | 1.15 | 1.11 | 1.19 | <0.001 |
| Cataract | 133.18 | 117.67 | 1.15 | 1.11 | 1.20 | <0.001 |
| Coronary heart disease | 114.87 | 101.44 | 1.15 | 1.10 | 1.20 | <0.001 |
| Atrial fibrillation | 184.43 | 164.33 | 1.15 | 1.09 | 1.22 | <0.001 |
| Diabetes mellitus | 152.18 | 135.64 | 1.14 | 1.10 | 1.18 | <0.001 |
| Hepatitis | 7.42 | 6.56 | 1.14 | 0.97 | 1.32 | 0.007 |
| Hyperthyroidism | 2.13 | 1.91 | 1.13 | 1.03 | 1.25 | <0.001 |
| Eating disorder | 19.60 | 17.31 | 1.13 | 0.85 | 1.50 | 0.168 |
| Vessel disease | 21.88 | 19.54 | 1.12 | 1.02 | 1.22 | <0.001 |
| Anxiety | 48.41 | 44.45 | 1.10 | 1.03 | 1.17 | <0.001 |
| Heart failure | 34.32 | 31.00 | 1.10 | 1.02 | 1.18 | <0.001 |
| Inflammatory bowel disease | 12.38 | 11.25 | 1.10 | 0.98 | 1.24 | 0.006 |
| Tuberculosis | 5.25 | 4.77 | 1.10 | 0.92 | 1.32 | 0.092 |
| Alcohol abuse | 18.20 | 16.76 | 1.09 | 0.99 | 1.20 | 0.004 |
| Chronic obstructive pulmonary disease | 93.35 | 86.95 | 1.08 | 1.03 | 1.13 | <0.001 |
| Tobacco abuse | 73.69 | 69.35 | 1.07 | 1.02 | 1.13 | <0.001 |
| Vision loss | 35.26 | 32.80 | 1.07 | 1.00 | 1.15 | 0.001 |
| Epilepsy | 11.35 | 10.70 | 1.06 | 0.94 | 1.20 | 0.118 |
| Chronic kidney disease | 44.87 | 42.49 | 1.05 | 0.98 | 1.12 | 0.017 |
| Solid malignancy | 98.69 | 98.27 | 1.00 | 0.96 | 1.04 | 0.929 |
| Peripheral vascular disease | 28.04 | 28.09 | 0.99 | 0.92 | 1.07 | 0.682 |
| Hematological malignancy | 7.22 | 7.64 | 0.94 | 0.81 | 1.10 | 0.182 |
| Parkinson's disease | 6.43 | 6.94 | 0.92 | 0.78 | 1.08 | 0.076 |
| Schizophrenia | 12.76 | 14.76 | 0.86 | 0.77 | 0.97 | <0.001 |
| Dementia | 10.56 | 12.87 | 0.80 | 0.71 | 0.91 | <0.001 |
| HIV positive / AIDS | 0.55 | 0.79 | 0.71 | 0.41 | 1.21 | 0.034 |
| Multiple sclerosis | 1.99 | 3.01 | 0.66 | 0.50 | 0.88 | <0.001 |
