# Supplementary material for: Comorbidity in incident osteoarthritis cases and matched controls using electronic health record data
Source: Arthritis Res Ther. 2023 Jul 4;25:114. doi: 10.1186/s13075-023-03086-8 (PMC10318652; doi:10.1186/s13075-023-03086-8)
Supplement: Supplementary file 3 — Additional file 3: Supplementary Table 3. Results of the sensitivity analysis: age & sex adjusted odds ratios with 99.9% confidence intervals and P-values of all comorbidities, assessed within the period of 1 year prior to the index date. [file 13075_2023_3086_MOESM3_ESM.docx]

Supplementary table 3. Results of the sensitivity analysis: age & sex adjusted odds ratios with 99.9% confidence intervals and P-values of all comorbidities, assessed within the period of 1 year prior to the index date

| Comorbidity | OR | Lower 99.9% CI | Upper 99.9% CI | P-value |
| --- | --- | --- | --- | --- |
| Fibromyalgia | 1.62 | 1.22 | 2.15 | <0.001 |
| Obesity | 1.27 | 1.12 | 1.43 | <0.001 |
| Chronic fatigue syndrome | 1.23 | 0.68 | 2.23 | 0.253 |
| Gout | 1.20 | 1.05 | 1.36 | <0.001 |
| Rheumatoid arthritis | 1.19 | 1.01 | 1.41 | <0.001 |
| Polymyalgia rheumatica | 1.14 | 0.86 | 1.51 | 0.130 |
| Spinal disc herniation | 1.13 | 1.04 | 1.22 | <0.001 |
| Thromboembolic disease | 1.00 | 0.87 | 1.15 | 0.993 |
| Neck pain | 0.99 | 0.92 | 1.08 | 0.749 |
| Sleeping disorder | 0.96 | 0.88 | 1.05 | 0.148 |
| Asthma | 0.95 | 0.84 | 1.08 | 0.188 |
| Liver cirrhosis | 0.95 | 0.75 | 1.21 | 0.492 |
| Hypothyroidism | 0.95 | 0.81 | 1.11 | 0.294 |
| Tuberculosis | 0.95 | 0.50 | 1.79 | 0.783 |
| Hearing loss | 0.94 | 0.85 | 1.04 | 0.034 |
| Osteoporosis | 0.93 | 0.84 | 1.03 | 0.027 |
| Back pain | 0.93 | 0.88 | 0.99 | <0.001 |
| Benin prostatic hyperplasia | 0.92 | 0.78 | 1.09 | 0.098 |
| Depression | 0.90 | 0.79 | 1.03 | 0.008 |
| Psoriasis | 0.89 | 0.75 | 1.06 | 0.027 |
| Atrial fibrillation | 0.89 | 0.79 | 0.99 | <0.001 |
| Allergy | 0.88 | 0.81 | 0.96 | <0.001 |
| Cataract | 0.88 | 0.82 | 0.94 | <0.001 |
| Eczema | 0.87 | 0.82 | 0.93 | <0.001 |
| Gastroesophageal reflux disease | 0.87 | 0.79 | 0.96 | <0.001 |
| Vertigo | 0.86 | 0.80 | 0.92 | <0.001 |
| Coronary heart disease | 0.86 | 0.78 | 0.95 | <0.001 |
| Vessel disease | 0.85 | 0.70 | 1.04 | 0.007 |
| Gallbladder disease | 0.85 | 0.74 | 0.98 | <0.001 |
| Hypertension | 0.85 | 0.80 | 0.90 | <0.001 |
| Heart failure | 0.83 | 0.73 | 0.96 | <0.001 |
| Chronic kidney disease | 0.82 | 0.74 | 0.91 | <0.001 |
| Anemia | 0.82 | 0.74 | 0.91 | <0.001 |
| Diabetes mellitus | 0.82 | 0.74 | 0.91 | <0.001 |
| Urolithiasis | 0.82 | 0.68 | 0.98 | <0.001 |
| Migraine | 0.81 | 0.68 | 0.98 | <0.001 |
| Eating disorder | 0.81 | 0.32 | 2.06 | 0.462 |
| Hypercholesterolemia | 0.80 | 0.73 | 0.87 | <0.001 |
| Peripheral vascular disease | 0.80 | 0.68 | 0.94 | <0.001 |
| Drug abuse | 0.79 | 0.60 | 1.05 | 0.007 |
| Vision loss | 0.79 | 0.68 | 0.92 | <0.001 |
| Sinusitis | 0.77 | 0.60 | 0.99 | <0.001 |
| Cerebrovascular accident | 0.76 | 0.68 | 0.86 | <0.001 |
| Anxiety | 0.76 | 0.65 | 0.89 | <0.001 |
| Chronic obstructive pulmonary disease | 0.75 | 0.66 | 0.84 | <0.001 |
| Hyperthyroidism | 0.75 | 0.56 | 0.99 | <0.001 |
| Alcohol abuse | 0.75 | 0.58 | 0.96 | <0.001 |
| Dementia | 0.74 | 0.61 | 0.89 | <0.001 |
| Inflammatory bowel disease | 0.74 | 0.53 | 1.02 | 0.002 |
| Hepatitis | 0.73 | 0.44 | 1.21 | 0.040 |
| Parkinson's disease | 0.71 | 0.51 | 0.98 | <0.001 |
| Schizophrenia | 0.70 | 0.55 | 0.90 | <0.001 |
| Tobacco abuse | 0.69 | 0.61 | 0.78 | <0.001 |
| Epilepsy | 0.68 | 0.48 | 0.98 | <0.001 |
| Solid malignancy | 0.67 | 0.61 | 0.74 | <0.001 |
| Multiple sclerosis | 0.65 | 0.28 | 1.52 | 0.097 |
| Hematological malignancy | 0.54 | 0.38 | 0.77 | <0.001 |
| HIV positive / AIDS | 0.14 | 0.00 | 4.13 | 0.057 |
